# Endovascular Embolization of a Dissected External Carotid Artery Pseudoaneurysm in a Young Female with Neurofibromatosis Complicated by Preeclampsia

**DOI:** 10.1155/2019/6020393

**Published:** 2019-06-12

**Authors:** Sasha Lalla, Rajeev Seecheran, Valmiki Seecheran, Sangeeta Persad, Ronald Henry, Naveen Anand Seecheran

**Affiliations:** ^1^Advanced Cardiovascular Institute, Port of Spain, Trinidad And Tobago; ^2^University of the West Indies, St. Augustine, Trinidad And Tobago; ^3^North Central Regional Health Authority, Mt. Hope, Trinidad And Tobago

## Abstract

Carotid artery pseudoaneurysms are infrequently encountered in clinical practice. Major contributory etiologies include blunt trauma, infections, cystic medial necrosis, fibromuscular dysplasia, arteriosclerosis, and congenital abnormalities. The authors report an exceedingly rare case of a dissected external carotid artery pseudoaneurysm in a 26-year-old female patient with neurofibromatosis complicated by preeclampsia at 28-week period of gestation, safely and successfully treated by coil embolization.

## 1. Introduction

Neurofibromatosis type-1 (NF-1) is an autosomal dominant genetic disorder that results from a mutation of the neurofibromin one gene, located on chromosome 17 (17q11.2). It is a multisystemic disorder with complete penetrance and an estimated prevalence of 1 in 3500 births. The prevalence of NF-1-associated vascular lesions ranges from 0.4 to 6.4%, according to a previous clinical series [1].

The spectrum of vasculopathies includes stenoses, aneurysms, and arteriovenous malformations; involving the aortic, renal, mesenteric, carotid-vertebral, subclavian-axillary, iliofemoral, intracerebral, and coronary arteries. Renal artery involvement is the most common [1].

Due to the rarity of NF-1-associated vasculopathy, clinical characteristics and management strategies have not yet been well ascertained. The authors report an exceedingly rare case of a dissected carotid artery pseudoaneurysm in a 26-year-old female patient with neurofibromatosis complicated by preeclampsia at 28-week period of gestation, safely and successfully treated via an endovascular coil embolization technique.

## 2. Case Report

A 26-year-old female with a medical history of neurofibromatosis type-1 and 28-week gestational age complicated by preeclampsia was referred to the cardiovascular center for evaluation of an expanding, pulsatile, tender mass on the right neck. On admission, her vital signs reflected a hypertensive emergency with systolic blood pressures of 200s mmHg, tachycardia of 112 beats per minute, tachypnea of 20 breaths per minute with oxygen saturation of 98% on room air. On physical examination, there were several features consistent with her preexisting diagnosis of NF-1, which included multiple “*café-au-lait*” macules and neurofibromas throughout her chest and abdomen, with both axillary and inguinal freckling [2].

**Figure 1:**
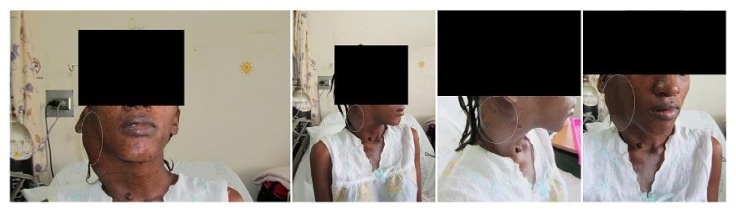
Facial depiction of the hematoma. Frontal series of the right neck hematoma (eclipsed in white), resulting from dissection of the right external carotid pseudoaneurysm.

**Figure 2:**
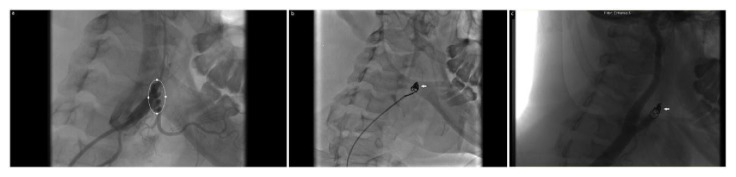
Right carotid artery cineangiography series. (a) Preintervention: the white ellipse encircles the external carotid artery dissection with contrast extravasation. (b) Intervention: The white arrow indicates successful coil embolization with 0.018” and 0.035” Tornado® embolization coils (Cook Medical® LLC, Bloomington, IN, USA). (c) Postintervention: the white arrow indicates complete occlusion of the external carotid artery distal to the superior thyroid branch; with no antegrade flow within the aneurysm.

Routine blood investigations were normal, 12-lead electrocardiography indicated a sinus tachycardia with left ventricular hypertrophy which was also visualized on an inpatient transthoracic echocardiogram. A computerized tomography scan revealed a dissected pseudoaneurysm of the right external carotid artery (ECA). In the interim, she was treated with an intravenous nitroglycerin infusion and hydralazine to achieve near-normotensive pressures over the ensuing 12 hours (see Figure 1). Subsequently, on the second day of hospitalization, selective carotid angiography demonstrated a dissected pseudoaneurysm of the right ECA measuring 2.7 cm, arising in association with the occipital branch with contrast extravasation (see Figure 2(a)). Ad-hoc successful coil embolization was achieved with 0.018” and 0.035” Tornado® embolization coils (Cook Medical LLC, Bloomington, IN, USA) (see Figure 2(b)). At the conclusion of the procedure, cineangiography revealed complete occlusion of the vessel distal to the superior thyroid branch with no further opacification of the aneurysm (see Figure 2(c)).

## 3. Discussion

NF-1 diagnostic criteria include the presence of two or more of the following criteria: six or more “*café-au-lait*” macules, two or more neurofibromas of any type, or one plexiform, neurofibroma; axillary or inguinal freckling; two or more Lisch nodules, optic pathway gliomas, distinctive bone lesions such as sphenoid dysplasia or thinning of the long bone cortex, with or without pseudoarthrosis, and a first degree relative diagnosed with NF-1 (see Table 1) [2].

ECA pseudoaneurysms are exceedingly rare within the general population as evidenced by the paucity of case reports, and consequently, optimal management strategies are not well established. The estimated prevalence is approximately 0.07% with a resultant mortality rate of 33% and only a handful of cases specifically involving the external carotid artery [3, 4].

Generally, these lesions accounted for only 1.4% of aneurysms in a recent review [5]. Intracranial aneurysms are also rare in cases with NF-1. Baldauf et al. identified 28 cases of intracranial aneurysms associated with neurofibromatosis predominantly located in the left internal carotid artery circulation with a female preponderance [6]. These clinical features were diametrically opposite in our patient as she displayed right external carotid artery involvement.

The pathophysiology of carotid pseudoaneurysm formation is usually attributed to penetrating or blunt trauma, usually from motor vehicle accidents. Other postulated mechanisms include direct compression of the neck, hyperextension/rotation injury and injury to the base of the skull, and anastomotic disruption following carotid vascular interventions [1]. Predisposing conditions include infection, radiotherapy, poor nutritional status, and neoplasia. Head and neck malignancies treated with radiotherapy can develop multifocal iatrogenic arteriopathy in the radiation field, leading to carotid “blow-out” [4, 7].

NF-1 is caused by a heterozygous mutation in the NF1 gene, which results in a loss of functional protein, neurofibromin, which is implicated in cell growth and differentiation. The mechanisms for the vasculopathy are complex and multifactorial, including smooth muscle dysplasia and direct vascular invasion by neurofibromatosis tissue [6]. This results in intimal proliferation of spindle cells with subsequent degenerative changes in the small vessel, including fibrosis, loss of the smooth muscle media, and elastin fragmentation. Additionally, in larger caliber vessels, neurofibromas or ganglioneuromas invade and weaken the arterial wall leading to aneurysm formation [8, 9].

The most common clinical presentation for NF-1 associated vasculopathy is renal artery stenosis with secondary hypertension [10–12]. Hypertension is present in 1% of NF1 patients and is significantly associated with mortality and morbidity [13]. Our patient did indeed present with a hypertensive crisis; however, renal artery stenosis was not diagnosed with Doppler ultrasonography.

Patients with dissected ECA pseudoaneurysms typically present with a tender, pulsatile neck mass. There may be an associated palpable thrill, audible bruit, or focal neurological deficits. Rapid enlargement of external carotid aneurysms may even result in rupture or cranial nerve entrapment [14, 15].

Diagnostic investigations include duplex ultrasonography with a sensitivity of 92%, with the significant caveat that it may incompletely visualize the distal internal carotid arteries. Computerized tomography imaging as a stand-alone modality may not be sensitive enough and often appears normal initially whereas digital subtraction angiography provides high-fidelity enhancement of the pseudoaneurysm, which may guide management strategies. Magnetic resonance angiography accurately demonstrates carotid dissection with sensitivity and specificity of 95% and 99%, respectively, as compared to 84% and 99% for magnetic resonance imaging alone [16].

Generally, the management of carotid pseudoaneurysms is not well delineated and more so focused on the internal carotid vasculature. Strategies include observation, anticoagulation, ligation of the carotid artery with or without a bypass procedure, and arterial reconstruction. With the advent and evolution of endovascular techniques, many periprocedural surgical complications such as cerebrovascular events (5-15%) and operative mortality (2-4%) have substantially decreased [17]. Of the transcatheter armamentarium, parent vessel occlusion with coil placement is currently in* vogue *with covered stent grafts becoming a viable alternative. In this clinical scenario, there was also the added complexity of the patient being of 28-week gestational age, and fetal exposure to radiation was a significant concern during fluoroscopy. A recent study indicated that the average radiation dose was circa 4100 mGy with fluoroscopy duration of 98 minutes and expended contrast media volume of approximately 130 mL [18]. The risk-benefit analysis of the alternative “open” vascular surgery and its attendant risks were considered, and, thus, the endovascular approach was favored. A systematic review indicated an endovascular procedural success of 92.8% with in-hospital mortality, stroke, and cranial nerve injury of 4.1%, 1.8%, and 0.5%, respectively. These results suggest that this approach has achieved comparable clinical outcomes to conventional surgery in patients with extracranial carotid artery aneurysms [17].

The outcome of carotid artery pseudoaneurysms is most aptly summarized by Winslow in 1926, who stated that “but it is not so much the rarity of this lesion, when it does occur that commands our attention as its propensity to imitate peritonsillar abscess, which habit of mimicry has lent on more than one occasion to lancing, with a mortal hemorrhage. The majority of the patients should recover if an aneurysm is promptly recognized and treated, but an overwhelming proportion dies under dilatory, blundering or pernicious tactics.” [19].

## 4. Conclusion

ECA pseudoaneurysms are exceedingly rare and should be considered when a patient presents with a tender, pulsatile neck mass. Early diagnostic imaging and invasive management should be immediately pursued with a high index of clinical suspicion as illustrated in this case, where endovascular embolization proved to be a safe and effective treatment modality.

## Figures and Tables

**Table 1:** Clinical criteria for Neurofibromatosis Type-1 (NF-1): ≥ 2 or more of the following [2].

≥ 6 “*café-au-lait*” macules
≥ 2 neurofibromas of any type or 1 plexiform neurofibroma
axillary or inguinal freckling
optic pathway gliomas
≥ 2 Lisch nodules
distinctive bone lesions such as sphenoid dysplasia or thinning of the long bone cortex,
with or without pseudoarthrosis
first-degree relative diagnosed with NF-1

## Data Availability

All available data can be obtained by contacting the corresponding author.

## Additional Points

*Key Clinical Message*. The clinician should be cognizant that a pulsatile neck mass can be a dissected aneurysm in a patient with neurofibromatosis type-1 which can be safely and successfully treated via endovascular embolization.
